# Pooled Safety Analysis of IncobotulinumtoxinA in the Treatment of Neurological Disorders in Adults

**DOI:** 10.3390/toxins15060353

**Published:** 2023-05-23

**Authors:** Wolfgang H. Jost, Petr Kaňovský, Michael A. Hast, Angelika Hanschmann, Michael Althaus, Atul T. Patel

**Affiliations:** 1 Parkinson-Klinik Ortenau, 77709 Wolfach, Germany; 2 Faculty of Medicine and Dentistry and University Hospital, Palacký University Olomouc, 779 00 Olomouc, Czech Republic; petr.kanovsky@fnol.cz; 3 Merz Pharmaceuticals, LLC, Raleigh, NC 27615, USA; michael.hast@merz.com; 4 Merz Therapeutics GmbH, 60318 Frankfurt am Main, Germany; angelika.hanschmann@merz.de (A.H.); michael.althaus@merz.de (M.A.); 5 Kansas City Bone and Joint Clinic, Overland Park, KS 66211, USA; apatel@kcbj.com

**Keywords:** incobotulinumtoxinA, cervical dystonia, blepharospasm, spasticity, sialorrhea, essential tremor, neurological disorders, motor disorders, safety, immunogenicity

## Abstract

The pooled incidences of treatment-emergent adverse events (TEAEs) were examined by indication using the integrated clinical database of Merz-sponsored, placebo-controlled, or repeat-dose studies of incobotulinumtoxinA in adults with cervical dystonia, blepharospasm, limb spasticity, sialorrhea, or essential tremor of the upper limb. Overall incidences of TEAEs, serious TEAEs, TEAEs leading to discontinuation, fatal TEAEs, TEAEs of special interest (TEAESIs; indicating possible toxin spread), and treatment-related (TR) events were determined for incobotulinumtoxinA and placebo after a single injection and for repeated dose cycles of incobotulinumtoxinA. The most frequent events after a single dose of incobotulinumtoxinA are summarized. After a single cycle, incidences of overall TEAEs were similar between incobotulinumtoxinA and the placebo in most indications, although between-indication differences were observed. Few TEAEs led to incobotulinumtoxinA discontinuation; there were no fatal TEAEs with incobotulinumtoxinA. In general, repeated cycles did not increase the incidence of any event. The most frequent TR-TEAEs were indication-dependent, including dysphagia for indications affecting the head or neck. The TR-TEAESIs across all indications were most commonly muscular weakness, dysphagia and dry mouth. Overall, the results of this pooled analysis support and extend the favorable safety and tolerability profile of incobotulinumtoxinA for the treatment of adult neurological disorders established by individual clinical studies.

## 1. Introduction

IncobotulinumtoxinA (Xeomin; Merz Pharmaceuticals GmbH, Frankfurt am Main, Germany) is a formulation of botulinum toxin type A (BoNT-A) that is purified to contain only the active neurotoxin and no accessory proteins or other bacterial proteins [1]. It is approved for use in adults in the United States for the treatment of cervical dystonia, blepharospasm, upper limb spasticity and chronic sialorrhea [1] and in Europe for cervical dystonia of a predominantly rotational form (spasmodic torticollis), blepharospasm, and hemifacial spasm, spasticity of the upper limb, and chronic sialorrhea due to neurological disorders [2]. Multiple clinical studies support the safety and efficacy of incobotulinumtoxinA in these indications [3,4,5,6,7,8,9,10,11,12,13,14,15,16,17,18,19,20], as well as evaluating the safety and efficacy of the biologic in subjects with lower limb spasticity [21] and essential tremor of the upper limb [22]. Additional indications, including some for pediatric populations, are also approved but are not the focus of this analysis [1,2].

As with any treatment, the nature and incidence of adverse events (AEs) can vary by trial and indication. A comprehensive, pooled assessment of data across the studies for these indications would provide additional insights into the safety of incobotulinumtoxinA and could identify any differences in the frequency of AEs across indications. Such an analysis has been conducted using incobotulinumtoxinA to treat different types of facial lines (glabellar frown lines, crow’s feet, and upper facial lines) [23]. That analysis revealed differences in the AE profile of the biologic across these indications and a possible decrease in the frequency of AEs with repeated injection cycles. 

The objective of this analysis was to further assess the incidence of treatment-related AEs across Merz-sponsored prospective placebo-controlled or repeat-dose incobotulinumtoxinA studies in adult subjects in cervical dystonia, blepharospasm, upper limb spasticity, lower limb spasticity, sialorrhea, and essential tremor of the upper limb. The studies included in these analyses all pre-dated the COVID-19 pandemic.

## 2. Results

The patient demographics varied by indication and study (Table 1). Similarly, the dose of incobotulinumtoxinA was indication-specific, being lowest in patients receiving treatment for blepharospasm and highest for those with spasticity. Across all studies, the planned duration of follow-up ranged from 6 to 121 weeks (Table 1).

### 2.1. Safety

#### 2.1.1. Overall Frequency of Adverse Events

After a single dose, the overall occurrence of treatment-emergent AEs (TEAEs) did not differ greatly between incobotulinumtoxinA and the placebo in most indications, although between-indication differences were observed (Table 2). In general, the incidences of TEAEs and TEAEs of special interest (TEAESIs; listed in Table 3), including those that were treatment-related, were highest in subjects receiving incobotulinumtoxinA or placebo for cervical dystonia or blepharospasm. Subjects receiving treatment for these indications also showed the greatest differences between incobotulinumtoxinA and the placebo in the overall incidences of TEAEs, particularly treatment-related TEAEs. Few serious TEAEs (SAEs) or TEAEs leading to discontinuation of incobotulinumtoxinA occurred, and only one subject each, both of whom were receiving on-label treatment only for lower limb spasticity, experienced a treatment-related SAE or a treatment-related TEAE that led to discontinuation of treatment. There were no fatal TEAEs in subjects receiving incobotulinumtoxinA. 

**Figure 1:**
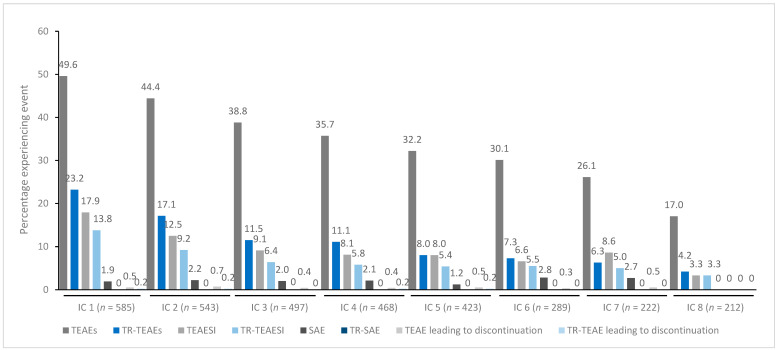
TEAE incidence by treatment cycle in patients with cervical dystonia treated with incobotulinumtoxinA. Note: In addition, 15 patients received a ninth IC (*n* = 2 TR-TEAE, *n* = 1 TR-TEAESI, no other events), eight patients received a tenth IC (*n* = 2 TEAE, *n* = 1 SAE, no other events), five patients received an eleventh IC (*n* = 1 TEAE, no other events), and one patient received a twelfth and thirteenth IC (no events). IC, injection cycle; INCO, incobotulinumtoxinA; TEAE, treatment-emergent adverse event; TEAESI, treatment-emergent adverse event of special interest (potentially indicative of toxin spread); SAE, serious TEAE; TR, treatment-related.

**Figure 2:**
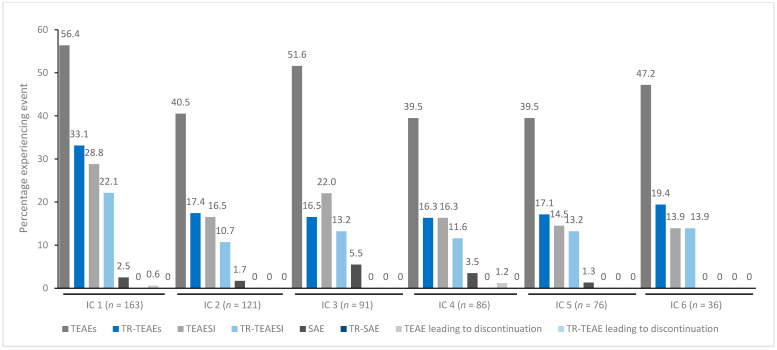
TEAE incidence by treatment cycle in patients with blepharospasm treated with incobotulinumtoxinA. IC, injection cycle; INCO, incobotulinumtoxinA; TEAE, treatment-emergent adverse event; TEAESI, treatment-emergent adverse event of special interest (potentially indicative of toxin spread); SAE, serious TEAE; TR, treatment-related.

**Figure 3:**
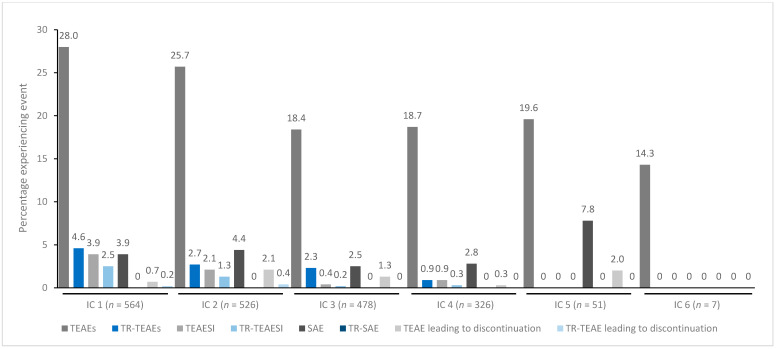
TEAE incidence by treatment cycle in patients with UL spasticity treated with incobotulinumtoxinA. IC, injection cycle; INCO, incobotulinumtoxinA; TEAE, treatment-emergent adverse event; TEAESI, treatment-emergent adverse event of special interest (potentially indicative of toxin spread); SAE, serious TEAE; TR, treatment-related; UL, upper limb.

**Figure 4:**
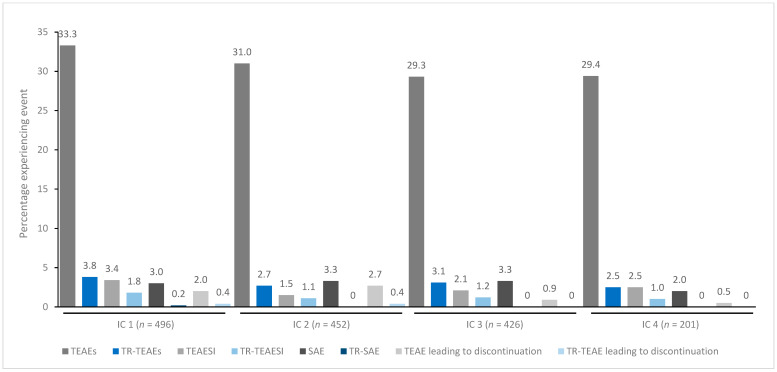
TEAE incidence by treatment cycle in patients with LL spasticity treated with incobotulinumtoxinA. IC, injection cycle; INCO, incobotulinumtoxinA; LL, lower limb; TEAE, treatment-emergent adverse event; TEAESI, treatment-emergent adverse event of special interest (potentially indicative of toxin spread); SAE, serious TEAE; TR, treatment-related.

**Figure 5:**
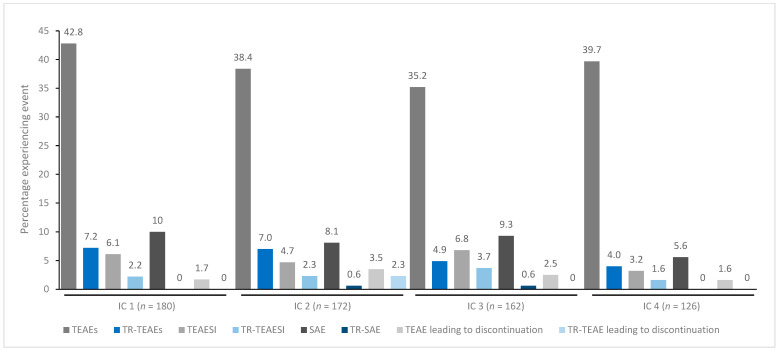
TEAE incidence by treatment cycle in patients with sialorrhea treated with incobotulinumtoxinA. IC, injection cycle; INCO, incobotulinumtoxinA; TEAE, treatment-emergent adverse event; TEAESI, treatment-emergent adverse event of special interest (potentially indicative of toxin spread); SAE, serious TEAE; TR, treatment-related.

In general, treatment for up to eight repeated cycles did not increase the incidences of any TEAE category (Figure 1, Figure 2, Figure 3, Figure 4 and Figure 5). Although some subjects who received incobotulinumtoxinA for cervical dystonia received additional cycles (up to 13), subject numbers were very small (≤15) relative to earlier cycles; therefore, analyses for these would not lead to meaningful results.

#### 2.1.2. Most Common Adverse Events after a Single-Dose

After a single dose, the most frequent TEAEs and treatment-related TEAEs were indication-dependent (Table 4, Table 5, Table 6, Table 7, Table 8 and Table 9), although injection site pain was reported by a small proportion of subjects across the indications. The most frequently reported TEAEs in subjects with cervical dystonia included dysphagia (in 15.7% of those treated with incobotulinumtoxinA and 4.1% of those who received a placebo), neck pain (10.1% vs. 4.1%), and muscular weakness (8.8% vs. 1.4%). These were also the most frequently reported treatment-related TEAEs in subjects with cervical dystonia (Table 4). In subjects with blepharospasm, eyelid ptosis (16.5% vs. 3.7%), dry eye (12.2% vs. 11.1%), and dry mouth (9.6% vs. 1.9%) were the most frequently reported TEAEs and treatment-related TEAEs (Table 5). 

In subjects with upper limb spasticity, nasopharyngitis was the most frequently reported TEAE (in 4.6% of incobotulinumtoxinA- and 0.9% of placebo-treated subjects), whereas dry mouth was the most frequent treatment-related TEAE (in 0.9% vs. 0.5% of subjects; Table 6). Similarly, in patients with lower limb spasticity, nasopharyngitis was the most frequently reported TEAE (6.5% vs. 4.8%), but muscular weakness was the most frequently reported treatment-related TEAE (Table 7).

Falling (5.4% vs. 2.8%) and dry mouth (4.7% vs. 0) were the most frequently reported TEAEs in subjects treated for sialorrhea, with dry mouth being the most frequent treatment-related TEAE (4.1% vs. 0; Table 8). The number of patients treated for essential tremor of the upper limb was small (*N* = 30 in one single-dose study), limiting the conclusions that could be drawn. In these subjects, muscular weakness was a common TEAE and treatment-related TEAE (Table 9).

In general, TEAESIs across all indications were most frequently muscular weakness, dysphagia, and/or dry mouth (Table 4, Table 5, Table 6, Table 7, Table 8 and Table 9). Similarly, these events were also the most frequent treatment-related TEAESIs, although subjects with blepharospasm also reported eyelid ptosis and blurred vision, and those with upper limb spasticity reported dysarthria.

### 2.2. Immunogenicity

In patients enrolled in repeat-dose studies, which were reported as treatment-naïve at baseline (*n* = 815), there was no observable pattern of hemidiaphragm assay (HDA) positivity at the study end (Table 10). Of the 20 patients who were treatment-naïve at baseline and were HDA positive at the study end, 50% were HDA positive at screening, and an additional 5% had a positive fluorescence immunoassay (FIA; but a missing HDA test) at screening. HDA positivity appeared more frequently in patients treated for spasticity or cervical dystonia (Table 10). The vast majority of positive HDA values were only marginally above the threshold of positivity, and the evolution of HDA values in individual patients did not correlate with the investigator’s judgment of efficacy, with no patients demonstrating a secondary lack of treatment response due to neutralizing antibodies (NAb).

## 3. Discussion

In this pooled analysis of placebo-controlled trials reporting data across a variety of neurological disorders in adults from a range of countries, incobotulinumtoxinA had a good safety profile, with no new or unexpected safety findings, no fatalities, and few SAEs. This analysis has helped to define and allow comparison of the safety profile of incobotulinumtoxinA administered over a range of doses for up to 13 injection cycles in subjects with cervical dystonia, blepharospasm, upper limb spasticity, lower limb spasticity, sialorrhea, and essential tremor of the upper limb. 

Findings were consistent with those of the individual trials included in this analysis [3,4,5,6,7,8,9,11,12,13,14,15,16,17,21,22], with TEAEs generally specific to each indication. The most common TEAEs by indication reported for incobotulinumtoxinA were comparable to those reported for all available BoNT-A formulations. Reviews of botulinum toxin therapy reveal that dysphagia, weakness, and upper respiratory complications are the main TEAEs to occur during the treatment of cervical dystonia; ptosis (usually temporary and self-resolving), vision changes, and dry eye are the most common complications of treatment of blepharospasm; viscous saliva, dry mouth, and dysphagia can occur with treatment for sialorrhea; and the treatment of spasticity can result in weakness or paralysis of off-target musculature [24,25,26,27,28,29,30,31,32,33,34,35,36,37,38,39,40,41,42,43,44,45,46,47,48,49,50,51]. There is potential for off-target muscle weakness or paralysis to lead to falls. However, our findings agree with the broader literature that demonstrates BoNT-A is well tolerated [24,25,52,53] and, when correctly administered, is considered very safe [54].

Analysis of up to eight repeated cycles of incobotulinumtoxinA revealed that, although fluctuations were observed, the incidences of all TEAE categories trended downwards over time. These findings are consistent with those of Coleman et al. [23], who confirmed the favorable safety and tolerability of incobotulinumtoxinA in patients with facial lines. In that analysis, investigators found that the frequency of treatment-related AEs with incobotulinumtoxinA was low and generally decreased with repeated injection cycles.

IncobotulinumtoxinA was associated with a very low rate of NAb formation over up to 13 injection cycles in the subjects evaluated, many of whom had received previous treatment with a botulinum toxin, and no patient demonstrated a secondary lack of treatment response due to NAb. Low rates of NAb formation have also been observed in a pooled analysis of clinical trials conducted in children with upper or lower limb spasticity or sialorrhea [55]. A recent cross-sectional study in 465 patients receiving either incobotulinumtoxinA or a complex protein-containing BoNT-A formulation (abobotulinumtoxinA or onabotulinumtoxinA) for periods of up to 10 years revealed that incobotulinumtoxinA reduces the risk of NAb induction compared with the complex protein-containing BoNT-A formulations [56], which may translate into a lower risk of secondary non-response with incobotulinumtoxinA. The findings of the current analyses showed the highest rates of positive HDA tests in patients with cervical dystonia or lower limb spasticity, and the lowest rates in those with blepharospasm or sialorrhea. Similar differences have been seen with other BoNT-A formulations [57,58,59,60,61] and, in part, may be related to the toxin dose administered [61,62,63,64] or the site of injection; for example, lymph node-rich regions, such as the neck, are more likely to produce an immune response [54,65]. Patients receiving treatment for sialorrhea or, in particular, blepharospasm also generally received lower doses of incobotulinumtoxinA than patients receiving treatment for the other indications. 

This analysis has limitations in that data for some indications were derived from single studies only, and the numbers of eligible subjects varied between indications. Nevertheless, findings were generally consistent across indications. Other limitations are that we could not analyze whether the development of TEAEs was related to injection technique and, in general, doses administered in the clinical trials included do not reflect the wide variability of dosing used in real-world clinical practice. Similarly, the follow-up duration (up to 104 weeks) is short relative to the required duration of treatment for many subjects in the real world, limiting the interpretation of both long-term safety and the immunogenicity data. Finally, all data were obtained from Merz-sponsored trials, which may have introduced some bias. It is possible that the administration of incobotulinumtoxinA in the controlled setting of a clinical trial may have reduced the risk for some TEAEs, but conversely, such settings increase the chances that all TEAEs will be collected.

## 4. Conclusions

Overall, the results of this pooled analysis support and extend the favorable safety and tolerability profile of incobotulinumtoxinA for the treatment of adult neurological disorders established by individual clinical studies.

## 5. Materials and Methods

Studies were obtained from the integrated clinical database containing data from all Merz-sponsored clinical trials of incobotulinumtoxinA (Supplementary Figure S1). The studies included were those that were placebo-controlled or were repeat-dose studies of incobotulinumtoxinA in adults with cervical dystonia, blepharospasm, upper limb spasticity, lower limb spasticity, sialorrhea, or essential tremor of the upper limb (Table 1). 

Single-dose and repeat-dose data were considered separately, although individual studies contributed to either or both analyses, depending on their design. Single-dose data were defined as those obtained from a study with a single treatment of incobotulinumtoxinA in a placebo-controlled setting or studies in which the first injection session of a repeat-dose study was placebo-controlled. Repeat-dose data were defined as those from studies in which subjects were intended to receive repeated treatments with incobotulinumtoxinA over ≥2 cycles; however, subjects who, for any reason, received only one incobotulinumtoxinA treatment in a repeat-dose study were also included in the safety analyses. Only subjects who received treatment with incobotulinumtoxinA were included in the repeat-dose analysis (i.e., subjects who received a placebo only were excluded).

### 5.1. Participants

All the participants were enrolled in the prospective clinical trials of incobotulinumtoxinA, summarized in Table 1. Subjects in all studies provided informed consent; all studies were institutional review board approved and conducted in accordance with the Declaration of Helsinki.

### 5.2. Data Extraction and Statistical Analysis

Subjects were pooled according to indication, and the single-dose and repeat-dose data were analyzed separately. The single-dose safety analysis set included all subjects who received one cycle of study medication or placebo. The repeat-dose safety analysis set included all subjects who received at least one cycle of study medication in a study that evaluated repeated doses of incobotulinumtoxinA (patients initially treated with placebo who subsequently received incobotulinumtoxinA in an open-label extension phase were included with the first dose of incobotulinumtoxinA considered injection cycle 1).

The incidence of TEAEs was summarized descriptively using counts and percentages; calculations were performed using statistical analysis system software (SAS) version 9.4 (SAS Institute, Cary, NC, USA). All TEAEs were coded according to the Medical Dictionary for Regulatory Activities (MedDRA) dictionary version 22.0. 

Specific analyses for single-dose data included: the overall incidence of TEAEs and the categories of SAEs, TEAEs leading to discontinuation, fatal TEAEs, as well as treatment-related events in all categories; the most frequent TEAEs (≥2% frequency in any group) and the most frequent treatment-related TEAEs (≥2 subjects in any group) were also summarized. TEAESIs, defined by regulatory authorities as potentially indicating toxin spread (Table 3), and treatment-related TEAESIs, were also analyzed for the single-dose data. In each study, subjects were asked to report TEAESIs, and in most studies, detailed active questioning for symptoms was used. 

For the repeat-dose data, the overall frequency of TEAEs, treatment-related TEAEs and SAEs were analyzed according to the treatment cycle.

NAb testing was also performed in most repeat-dose studies and is reported for all subjects who received at least one cycle of incobotulinumtoxinA across all studies with testing. In general, antibody samples were collected before the first treatment injection at the screening or baseline visit and the final individual visit of each trial. Blood samples for immunogenicity testing were screened using FIA to detect any binding antibodies against botulinum toxin in the first step. In case of a positive FIA finding, further testing with the highly sensitive mouse ex vivo HDA for the final confirmation of NAb presence and respective determination of titers was performed as a second step. Descriptive results are presented with no statistical testing. Immunogenicity data were evaluated by indication and previous treatment (previously treated—defined as pre-treatment with botulinum toxin for any indication before study entry—or treatment naïve).

## Figures and Tables

**Table 1:** Summary of prospective clinical trials included in the safety analysis of incobotulinumtoxinA in patients with neurological disorders.

	Reference	Study Design	SD vs. RD	No. of Patients ^a^	Baseline Patient Characteristics (Study Location)	Treatments	Duration
Cervical dystonia	NCT00407030 (Comella et al., 2011 [3]; Evidente et al. 2013 [5])	Double-blind, randomized, placebo-controlled, multicenter	SD	233	66% female; mean age 52.8 years; mean duration of CD 51.9 months; 91% White; 61% BoNT pre-treated (US)	INCO 120 U INCO 240 U Placebo	≤20 weeks
		MP + double-blind, randomized, multicenter extension	RD	227		INCO 120 U INCO 240 U	≤5 ICs
	NCT01486264 (Comella et al., 2022 [4])	Open-label, randomized, non-inferiority, multicenter	RD	282	72% female; mean age 57.1 years; 91% White; 100% BoNT pre-treated for CD (≥3 previous BoNT injections within 52 weeks) (US)	INCO short IC (q8w) INCO standard IC (q14ws)	≤8 ICs ≤68/104 weeks
	NCT00541905 Dressler et al. (2013 [6])	Multicenter, open-label, single-arm	RD	76	66% female; mean age 54.4 years; mean duration of CD 9.2 years; 99% White; 75% BoNT pre-treated (Germany)	INCO ≤ 300 U q10–24 weeks	5 ICs ≤121 weeks
Blepharospasm	NCT00406367 Jankovic et al., 2011 [7]; Truong et al., 2013 [8])	Double-blind, randomized, placebo-controlled	SD	108	65% female; mean age 61.9 years; 82% White; 100% BoNT pre-treated (US)	INCO ≤ 50 U/eye Placebo	≤20 weeks
		MP + open-label extension	RD	106		INCO ≤ 50 U/eye	+≤5 ICs (+≤49 weeks) + safety observation 20 weeks
	NCT01896895 (Mitsikostas et al., 2020 [9])	Double-blind, randomized, placebo-controlled	SD	61	59% female; mean age 55.0 years; 23% White, 77% Asian; 100% no BoNT pre-treatment in prior year (Greece, Malaysia, Sri Lanka)	INCO 12.5 U/eye INCO 25 U/eye Placebo	6–20 weeks
		MP + open-label extension	RD	57		INCO ≤ 35 U/eye	+1 IC +6–20 weeks
UL spasticity	NCT00432666 (Kaňovský et al., 2009 [11], 2011 [12])	Double-blind, randomized, placebo-controlled, multicenter, Phase 3	SD	148	36% female; mean age 55.6 years; 100% White; 24% BoNT pre-treated (Czech Republic, Hungary, Poland)	INCO ≤ 400 U Placebo	≤20 weeks
		MP + open-label extension	RD	147		INCO ≤ 400 U	+≤5 ICs +≤69 weeks
	NCT01392300 (Elovic et al., 2016 [13], Marciniak et al., 2019 [14])	Double-blind, randomized, placebo-controlled, multicenter	SD	317	43% female; mean age 56.1 years; 84% White, 13% Asian; 17% BoNT pre-treated (Czech Republic, Germany, Hungary, India, Poland, Russian Federation, US)	INCO 400 U Placebo	12 weeks
		MP + open-label extension	RD	309		INCO 400 U q12w	+≤3 ICs +≤36 weeks
	JapicCTI-153029 (Masakado et al., 2020 [15])	Double-blind, randomized, placebo-controlled, multicenter, Phase 3	SD	100	25% female; mean age 59.7 years; 100% Asian; 80% BoNT pre-treated (Japan)	INCO 250 U INCO 400 U Placebo	12 weeks
		MP + open-label extension	RD	108		INCO 400 U	+≤3 ICs +≤40 weeks
LL spasticity	NCT01464307	Double-blind, randomized, placebo-controlled, multicenter, Phase 3	SD	289	33% female; mean age 57.2 years; 96% White; 26% BoNT pre-treated (Canada, Czech Republic, France, Germany, Italy, Poland, Russian Republic, Spain, US)	INCO 400 U Placebo	12 weeks
		MP + open-label extension	RD	284		INCO 400 U	
	JapicCTI-153030 (Masakado et al., 2022 [21])	Double-blind, randomized, placebo-controlled, multicenter, Phase 3	SD	208	24% female; mean age 59.2 years; 100% Asian; 52% BoNT pre-treated (Japan)	INCO 400 U Placebo	12 weeks
		MP + open-label extension	RD	212		INCO 400 U	+≤3 ICs +≤40 weeks
Sialorrhea	NCT02091739 (Jost et al., 2019 [16], 2020 [17])	Double-blind, randomized, placebo-controlled, multicenter, Phase 3	SD	184	29% female; mean age 65.2 years; etiology: Parkinson’s disease 70.7%, stroke 17.9%, atypical parkinsonism 8.7%, TBI 2.7%; 99% White; 100% BoNT pre-treatment status missing (Germany, Poland)	INCO 75 U INCO 100 U Placebo	16 weeks
		MP + open-label extension	RD	180		INCO 75 U q16w INCO 100 U q16w	+≤3 ICs +≤48 weeks
Essential tremor of the UL	NCT02207946 (Jog et al., 2020 [22])	Double-blind, randomized, placebo-controlled, multicenter, Phase 2	SD	30	50% female; mean age 68.1 years; 93% White; 100% BoNT pre-treatment status missing (Canada, US)	INCO 30–200 UPlacebo	24 weeks

^a^ Number of patients from the study included in the current analyses. +, in addition to the SD period; BoNT, botulinum toxin (A or B); CD, cervical dystonia; IC, injection cycle; INCO, incobotulinumtoxinA; LL, lower limb; MP, main period (placebo-controlled injection cycle of incobotulinumtoxinA); qxw, every × weeks; RD, repeat-dose; SD, single-dose; TBI, traumatic brain injury; UL, upper limb, US, United States.

**Table 2:** Pooled incidence of TEAEs after a single dose in PBO-controlled studies evaluating incobotulinumtoxinA in the treatment of adults with neurological disorders by indication.

	Cervical Dystonia		Blepharospasm		UL Spasticity		LL Spasticity		Sialorrhea		ET of the UL	
No. of studies	1		2		3		2		1		1	
	INCO	PBO	INCO	PBO	INCO	PBO	INCO	PBO	INCO	PBO	INCO	PBO
No. of subjects	159	74	115	54	350	215	248	249	148	36	19	11
TEAE, *n* (%)	91 (57.2)	36 (48.6)	68 (59.1)	26 (48.1)	104 (29.7)	51 (23.7)	92 (37.1)	95 (38.2)	67 (45.3)	16 (44.4)	9 (47.4)	6 (54.5)
TR-TEAE, *n* (%)	58 (36.5)	12 (16.2)	43 (37.4)	9 (16.7)	15 (4.3)	5 (2.3)	10 (4.0)	14 (5.6)	13 (8.8)	3 (8.3)	3 (15.8)	1 (9.1)
SAE, *n* (%)	4 (2.5)	0	3 (2.6)	1 (1.9)	14 (4.0)	4 (1.9)	10 (4.0)	6 (2.4)	16 (10.8)	3 (8.3)	0	2 (18.2)
TR-SAE, *n* (%)	0	0	0	0	0	0	1 (0.4)	0	0	0	0	0
TEAE disc	0	0	1 (0.9)	0	2 (0.6)	4 (1.9)	8 (3.2)	4 (1.6)	2 (1.4)	1 (2.8)	0	1 (9.1)
TR-TEAE disc	0	0	0	0	0	0	1 (0.4)	0	0	0	0	0
TEAESI, *n* (%)	36 (22.6)	5 (6.8)	36 (31.3)	9 (16.7)	11 (3.1)	4 (1.9)	11 (4.4)	7 (2.8)	10 (6.8)	0	3 (15.8)	1 (9.1)
TR-TEAESI, *n* (%)	30 (18.9)	4 (5.4)	28 (24.3)	3 (5.6)	6 (1.7)	3 (1.4)	5 (2.0)	5 (2.0)	4 (2.7)	0	2 (10.5)	0
Fatal TEAE	0	0	0	0	0	1 (0.5)	0	1 (0.4)	0	0	0	0
Fatal TR-TEAE	0	0	0	0	0	0	0	0	0	0	0	0

disc, leading to discontinuation; ET, essential tremor; INCO, incobotulinumtoxinA; LL, lower limb; PBO, placebo; TEAE, treatment-emergent adverse event; TEAESI, treatment-emergent adverse event of special interest; TR, treatment-related; SAE, serious TEAE; UL, upper limb.

**Table 3:** Summary of adverse events of special interest that may potentially indicate toxin spread.

Accommodation Disorder	IIIrd Nerve Paresis
Areflexia	Ileus paralytic
Aspiration	IVth nerve paresis
Botulism	Monoparesis
Bradycardia	Muscular weakness
Bulbar palsy	Paralysis
Constipation	Paraparesis
Cranial nerve palsies multiple	Paresis
Cranial nerve paralysis	Paresis cranial nerve
Diaphragmatic paralysis	Peripheral nerve palsy
Diplopia	Peripheral paralysis
Dry mouth	Pelvic floor muscle weakness
Dysarthria	Pneumonia aspiration
Dysphagia	Pupillary reflex impaired
Dysphonia	Quadriparesis
Dyspnea	Respiratory arrest
Extraocular muscle paresis	Respiratory depression
Eyelid function disorder	Respiratory failure
Eyelid ptosis	Speech disorder
Facial paralysis	Trigeminal nerve paresis
Facial paresis	Urinary retention
Hemiparesis	Vision blurred
Hypoglossal nerve paresis	Vocal cord paralysis
Hyporeflexia	Vocal cord paresis
Hypotonia	

Adverse events of special interest were as specified by regulatory authorities and were identified using Medical Dictionary for Regulatory Activities (MedDRA) Version 22 preferred terms (or the most recent MedDRA version available at the time of the study).

**Table 4:** Summary of the most common treatment-emergent adverse events overall and of special interest in single-dose placebo-controlled studies of incobotulinumtoxinA in patients with cervical dystonia.

MedDRA Preferred Term	IncobotulinumtoxinA *N* = 159 *n* (%)	Placebo *N* = 74 *n* (%)
**TEAEs**		
Dysphagia	25 (15.7)	3 (4.1)
Neck pain	16 (10.1)	3 (4.1)
Muscular weakness	14 (8.8)	1 (1.4)
Injection site pain	11 (6.9)	4 (5.4)
Musculoskeletal pain	9 (5.7)	1 (1.4)
Headache	7 (4.4)	3 (4.1)
Nausea	6 (3.8)	0
Dizziness	5 (3.1)	1 (1.4)
Musculoskeletal stiffness	5 (3.1)	1 (1.4)
Sinusitis	5 (3.1)	2 (2.7)
Muscle spasms	4 (2.5)	2 (2.7)
Asthma	4 (2.5)	0
Oropharyngeal pain	4 (2.5)	2 (2.7)
**Treatment-related TEAEs**		
Dysphagia	22 (13.8)	3 (4.1)
Neck pain	14 (8.8)	1 (1.4)
Muscular weakness	13 (8.2)	1 (1.4)
Injection site pain	11 (6.9)	4 (5.4)
Musculoskeletal pain	8 (5.0)	0
Headache	4 (2.5)	0
Nausea	3 (1.9)	0
Dizziness	2 (1.3)	0
Musculoskeletal stiffness	5 (3.1)	1 (1.4)
Muscle spasms	3 (1.9)	1 (1.4)
Myalgia	3 (1.9)	0
Asthenia	2 (1.3)	0
Dry mouth	2 (1.3)	0
Head discomfort	2 (1.3)	0
Presyncope	2 (1.3)	0
Hyperhidrosis	2 (1.3)	0
**TEAESIs**		
Dysphagia	25 (15.7)	3 (4.1)
Muscular weakness	14 (8.8)	1 (1.4)
**Treatment-related TEAESI**		
Dysphagia	22 (13.8)	3 (4.1)
Muscular weakness	13 (8.2)	1 (1.4)
Dry mouth	2 (1.3)	0

Any TEAE/TEAESI occurring in >2% of incobotulinumtoxinA recipients, and any treatment-related TEAE/TEAESI occurring in ≥2 incobotulinumtoxinA recipients. MedDRA, Medical Dictionary for Regulatory Activities; TEAE, treatment-related adverse event; TEAESI, treatment-emergent adverse events of special interest.

**Table 5:** Summary of the most common treatment-emergent adverse events overall and of special interest in single-dose placebo-controlled studies of incobotulinumtoxinA in patients with blepharospasm.

MedDRA Preferred Term	IncobotulinumtoxinA *N* = 115 *n* (%)	Placebo *N* = 54 *n* (%)
**TEAEs**		
Eyelid ptosis	19 (16.5)	2 (3.7)
Dry eye	14 (12.2)	6 (11.1)
Dry mouth	11 (9.6)	1 (1.9)
Headache	7 (6.1)	2 (3.7)
Diarrhea	6 (5.2)	0
Vision impairment	6 (5.2)	0
Vision blurred	5 (4.3)	2 (3.7)
Dyspnea	5 (4.3)	1 (1.9)
Respiratory tract infections	5 (4.3)	1 (1.9)
Nasopharyngitis	4 (3.5)	2 (3.7)
Dysphagia	3 (2.6)	2 (3.7)
Asthenia	3 (2.6)	0
Injection site pain	3 (2.6)	0
**Treatment-related TEAEs**		
Eyelid ptosis	18 (15.7)	1 (1.9)
Dry eye	11 (9.6)	4 (7.4)
Dry mouth	7 (6.1)	1 (1.9)
Vision blurred	3 (2.6)	0
Injection site pain	3 (2.6)	0
Vision impairment	2 (1.7)	0
**TEAESI**		
Eyelid ptosis	19 (16.5)	2 (3.7)
Dry mouth	11 (9.6)	1 (1.9)
Vision blurred	5 (4.3)	2 (3.7)
Dyspnea	5 (4.3)	1 (1.9)
Dysphagia	3 (2.6)	2 (3.7)
**Treatment-related TEAESI**		
Eyelid ptosis	18 (15.7)	1 (1.9)
Dry mouth	7 (6.1)	1 (1.9)
Vision blurred	3 (2.6)	0

Any TEAE/TEAESI occurring in >2% of incobotulinumtoxinA recipients, and any treatment-related TEAE/TEAESI occurring in ≥2 incobotulinumtoxinA recipients. MedDRA, Medical Dictionary for Regulatory Activities; TEAE, treatment-related adverse event; TEAESI, treatment-emergent adverse events of special interest.

**Table 6:** Summary of the most common treatment-emergent adverse events overall and of special interest in single-dose placebo-controlled studies of incobotulinumtoxinA in patients with upper limb spasticity.

MedDRA Preferred Term	IncobotulinumtoxinA *N* = 350 *n* (%)	Placebo *N* = 215 *n* (%)
**TEAEs**		
Nasopharyngitis	16 (4.6)	2 (0.9)
**Treatment-related TEAEs**		
Dry mouth	3 (0.9)	1 (0.5)
Hypoesthesia	2 (0.6)	0
Dysarthria	2 (0.6)	0
**TEAESIs**	None reported with >2% incidence	None reported with >2% incidence
**Treatment-related TEAESIs**		
Dry mouth	3 (0.9)	1 (0.5)
Dysarthria	2 (0.6)	0

Any TEAE/TEAESI occurring in >2% of incobotulinumtoxinA recipients, and any treatment-related TEAE/TEAESI occurring in ≥2 incobotulinumtoxinA recipients. MedDRA, Medical Dictionary for Regulatory Activities; TEAE, treatment-related adverse event; TEAESI, treatment-emergent adverse events of special interest.

**Table 7:** Summary of the most common treatment-emergent adverse events overall and of special interest in single-dose placebo-controlled studies of incobotulinumtoxinA in patients with lower limb spasticity.

MedDRA Preferred Term	IncobotulinumtoxinA *N* = 248 *n* (%)	Placebo *N* = 249 *n* (%)
**TEAEs**		
Nasopharyngitis	16 (6.5)	12 (4.8)
Fall	8 (3.2)	5 (2.0)
Pain in extremity	7 (2.8)	8 (3.2)
Eczema	6 (2.4)	1 (0.4)
Muscular weakness	5 (2.0)	3 (1.2)
**Treatment-related TEAEs**		
Muscular weakness	3 (1.2)	3 (1.2)
**TEAESIs**		
Muscular weakness	5 (2.0)	3 (1.2)
**Treatment-related TEAESIs**		
Muscular weakness	3 (1.2)	3 (1.2)

Any TEAE/TEAESI occurring in >2% of incobotulinumtoxinA recipients, and any treatment-related TEAE/TEAESI occurring in ≥2 incobotulinumtoxinA recipients. MedDRA, Medical Dictionary for Regulatory Activities; TEAE, treatment-related adverse event; TEAESI, treatment-emergent adverse events of special interest.

**Table 8:** Summary of the most common treatment-emergent adverse events overall and of special interest in single-dose placebo-controlled studies of incobotulinumtoxinA in patients with sialorrhea.

MedDRA Preferred Term	IncobotulinumtoxinA *N* = 148 *n* (%)	Placebo *N* = 36 *n* (%)
**TEAEs**		
Fall	8 (5.4)	1 (2.8)
Dry mouth	7 (4.7)	0
Hypertension	5 (3.4)	1 (2.8)
Tooth extraction	4 (2.7)	0
Contusion	4 (2.7)	0
Diarrhea	4 (2.7)	1 (2.8)
Nasopharyngitis	4 (2.7)	1 (2.8)
Dysphagia	3 (2.0)	0
Bronchitis	3 (2.0)	0
Urinary tract infection	3 (2.0)	0
Parkinson’s disease	3 (2.0)	0
**Treatment-related TEAEs**		
Dry mouth	6 (4.1)	0
Dysphagia	2 (1.4)	0
Paresthesia	2 (1.4)	0
**TEAESIs**		
Dysphagia	3 (2.0)	0
**Treatment-related TEAESIs**		
Dysphagia	2 (1.4)	0

Any TEAE/TEAESI occurring in >2% of incobotulinumtoxinA recipients, and any treatment-related TEAE/TEAESI occurring in ≥2 incobotulinumtoxinA recipients. MedDRA, Medical Dictionary for Regulatory Activities; TEAE, treatment-related adverse event; TEAESI, treatment-emergent adverse events of special interest.

**Table 9:** Summary of the most common treatment-emergent adverse events overall and of special interest in single-dose placebo-controlled studies of incobotulinumtoxinA in patients with essential tremor of the upper limb.

MedDRA Preferred Term	IncobotulinumtoxinA *N* = 19 *n* (%)	Placebo *N* = 11 *n* (%)
**TEAEs**		
Upper respiratory tract infection	2 (10.5)	2 (18.2)
Muscular weakness	2 (10.5)	0
**Treatment-related TEAEs**		
Muscular weakness	2 (10.5)	0
Injection site bruising	1 (5.3)	0
Injection site pain	1 (5.3)	0
**TEAESIs**		
Muscular weakness	2 (10.5)	0
Dry mouth	1 (5.3)	0
Dysphonia	1 (5.3)	0
**Treatment-related TEAESIs**		
Muscular weakness	2 (10.5)	0

Any TEAE occurring in ≥2 incobotulinumtoxinA recipients/TEAESI occurring in ≥1 incobotulinumtoxinA recipients, and any treatment-related TEAE/TEAESI occurring in ≥1 incobotulinumtoxinA recipient. Note that the definition of common differs for this indication because of the small number of included subjects. MedDRA, Medical Dictionary for Regulatory Activities; TEAE, treatment-related adverse events; TEAESI, treatment-emergent adverse events of special interest.

**Table 10:** Summary of immunogenicity with incobotulinumtoxinA, overall and by botulinum toxin pre-treatment status.

	Study Screening			Last Study Visit ^a^		
Indication	*N* Tested	Positive FIA Test, *n* (%)	Positive HDA Test, *n* (%)	*N* Tested	Positive FIA Test, *n* (%)	Positive HDA Test, *n* (%)
Cervical dystonia	578	46 (8.0)	16 (2.8)	522	42 (8.0)	16 (3.1) ^b,c^
Blepharospasm	169	4 (2.4)	0 ^b^	162	7 (4.3)	1 (0.6)
UL spasticity	576	25 (4.3)	8 (1.4)	569	28 (4.9)	12 (2.1)
LL spasticity	507	28 (5.5)	12 (2.4) ^b^	506	31 (6.1)	18 (3.6) ^b^
Sialorrhea	128	0	0 ^c^	173	2 (1.2)	1 (0.6)
Overall	1958	103 (5.3)	36 (1.8) ^d^	1932	110 (5.7)	48 (2.5) ^d^
Treatment naïve ^e^	815	33 (4.0)	12 (1.5)	810	39 (4.8)	20 (2.5) ^b^
Pre-treated ^f^	953	66 (6.9)	24 (2.5) ^b^	891	64 (7.2)	26 (2.9) ^b^
Missing	190	4 (2.1)	0 ^b^	231	7 (3.0)	2 (0.9)

^a^ Last visit refers to an individual’s last visit, meaning that at least one post-baseline antibody measurement was available. ^b^ One additional patient had a borderline HDA result. This occurred because a single test could not unambiguously differentiate a true positive from a false positive test result. ^c^ One patient had an HDA measurement without having an FIA measurement at the same visit; this patient was HDA negative. ^d^ Two additional patients had a borderline HDA result. This occurred because a single test could not unambiguously differentiate a true positive from a false positive test result. ^e^ All patients who were treatment-naïve and had a positive HDA test at the last study visit were receiving incobotulinumtoxinA for UL or LL spasticity: 6 had negative FIA results at screening but an intermittent pattern of negative and positive HDA values during the study, being HDA-positive at their last study visit; in 4, the HDA was positive only at the last visit, but all other results throughout the study were negative; 10 had a positive HDA test at screening despite being treatment-naïve. ^f^ Pre-treatment was defined as pre-treatment with botulinum toxin for any indication at any time prior to study entry. FIA, fluorescence immunoassay; HDA, hemidiaphragm assay; LL, lower limb; UL, upper limb.

## Data Availability

Data are not publicly available.

## Supplementary Materials

The following supporting information can be downloaded at: https://www.mdpi.com/article/10.3390/toxins15060353/s1, Figure S1: Selection of clinical trials to be included in the pooled analysis.
