# Supplementary figures and images for: Pooled Safety Analysis of IncobotulinumtoxinA in the Treatment of Neurological Disorders in Adults
Source: Toxins (Basel). 2023 May 23;15(6):353. doi: 10.3390/toxins15060353 (PMC10301625; doi:10.3390/toxins15060353)

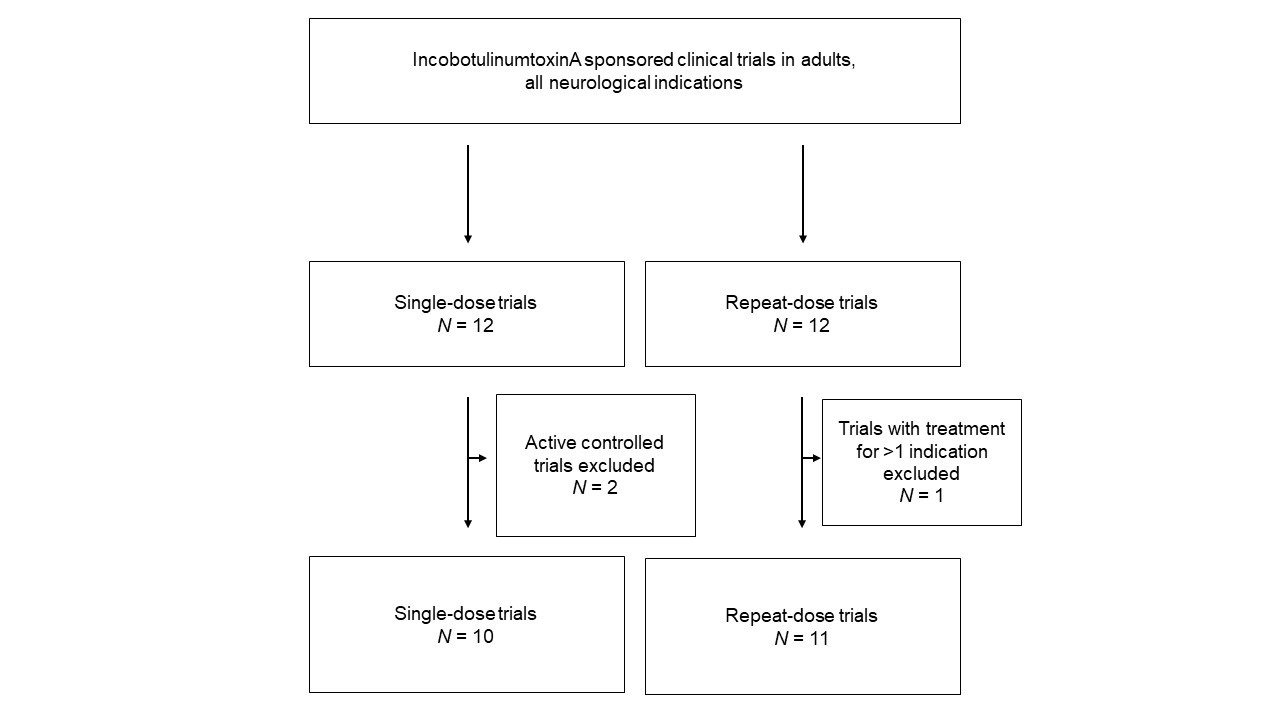

Supplement: Supplementary file 1 [file toxins-15-00353-s001.zip › toxins-2360393-supplementary.jpg]
